# A feasibility study with process evaluation of a teacher led resource to improve measures of child health

**DOI:** 10.1371/journal.pone.0218243

**Published:** 2019-07-02

**Authors:** Duncan S. Buchan, Samantha Donnelly, Gillian McLellan, Ann-Marie Gibson, Rosemary Arthur

**Affiliations:** 1 School of Health and Life Sciences, University of the West of Scotland, South Lanarkshire, Scotland, United Kingdom; 2 School of Psychological Sciences and Health, University of Strathclyde, Glasgow, Scotland, United Kingdom; IRCCS E. Medea, ITALY

## Abstract

Previous school-based interventions have produced positive effects upon measures of children’s health and wellbeing but such interventions are often delivered by external experts which result in short-term effects. Thus, upskilling and expanding the resources available to classroom teachers could provide longer-term solutions. This paper presents a feasibility study of an online health resource (Healthy Schools Resource: HSR) developed to assist primary school teachers in the delivery of health-related education. Four schools (*n* = 2 intervention) participated in this study. Study feasibility was assessed by recruitment, retention and completion rates of several outcomes including height, weight, waist circumference, blood pressure and several metabolic markers including HDL-cholesterol, triglycerides, glucose and dietary knowledge following a 10-12-week intervention period. The process evaluation involved fidelity checks of teachers’ use of the HSR and post-intervention teacher interviews. A total of 614 consent forms were issued and 267 were returned (43%), of which, 201 confirmed consent for blood sampling (75%). Retention of children participating in the study was also high (96%). Of the 13 teachers who delivered the intervention to the children, four teachers were excluded from further analyses as they did not participate in the fidelity checks. Overall, teachers found the online resource facilitative of teaching health and wellbeing and several recommendations regarding the resource were provided to inform further evaluations. Recruitment and retention rates suggest that the teacher led intervention is feasible and acceptable to both teachers, parents and children. Initial findings provide promising evidence that given a greater sample size, a longer intervention exposure period and changes made to the resource, teachers’ use of HSR could enhance measures of health and wellbeing in children.

## Introduction

Childhood obesity has been identified as one of the greatest global health problems of the 21^st^ century [1]. Childhood obesity levels are of particular concern in the UK, with approximately 28% of children aged 2-15-years from England and Scotland being overweight or obese [2,3]. Physical inactivity and poor dietary habits in childhood are known to be associated with greater levels of adiposity and poor cardiometabolic risk profiles which if left unabated, could track into adulthood [4]. Since children’s participation in physical activity (PA) tends to decline in adolescence [5,6] and poor dietary habits tend to follow similar trends from childhood into adulthood [7], it is important to develop, evaluate and identify potential efficacious interventions which can be introduced early to children to offset the likelihood of becoming physically inactive and developing poor dietary behaviours.

The school environment is considered an ideal setting to facilitate health interventions because of their pre-established infrastructure and the prolonged amount of time children spend there [8]. Indeed, evidence supports school-based interventions as a strategy to educate and improve measures of health and wellbeing in children [9–11]. Yet, systematic reviews which have evaluated the effects of school-based interventions designed to improve diet, physical activity or reduce the incidence of obesity suggest that their influence is limited [9,10]. Whereas previous school-based interventions comprising of PA and nutritional education components have proved successful in the short-term [12,13], evidence is scarce for supporting any long-term effects [9,14,15]. Moreover, as many school-based interventions require external experts or specialists on a short-term basis with little involvement of classroom teachers [11], it is unsurprising there is a dearth of long term evaluations given the questionable cost effectiveness and long term sustainability of such approaches.

One such long term evaluation by Kipping and colleagues [15] within the UK highlights the difficulty of positively influencing PA and dietary behaviours through school-based interventions. In this study a large cluster randomized controlled trial was conducted in 60 primary schools with the intervention arm of the cohort provided with 16 detailed lesson plans and 10 parent-child interactive components that were adapted from the Planet Health programme in the US [16]. Even though teachers trained in the resource delivered the lesson content over two out of the three school terms, the intervention failed to increase levels of PA, fruit and vegetable consumption or reduce sedentary behaviour [15]. Despite the limited effects, a notable strength of this study was the use of classroom teachers to deliver the lesson content and facilitate the intervention. Classroom teachers can be effective facilitators of school-based health interventions yet many face significant barriers including lack of time, support and training to effectively increase PA and improve the dietary habits of children [17,18]. Moreover, many teachers can feel overwhelmed when asked to implement health promotion initiatives on top of processing constant educational reforms within their curriculum [19]. Naturally, it has been suggested that if health initiatives are to be successful long-term, greater integration within the curriculum is essential [19,20].

In Scotland, the Curriculum for Excellence (CfE) was introduced in 2011 to assist teachers in the delivery of a holistic and learner centred approach to the curriculum [21]. With the move away from a central prescription of the curriculum to an approach which relies more upon the abilities of teachers to adapt curriculum guidance to meet the needs of their learners [22], teachers may struggle finding, understanding and then delivering curricular health and wellbeing education. Given these concerns, NHS Lanarkshire and local Education Authorities developed an online teacher resource to specifically address the need for a coordinated holistic approach to health and wellbeing curriculum delivery within nurseries and primary schools.

Developed as a cost and time efficient mean of supporting primary school teachers in Scotland, The Healthy Eating, Active Lifestyles Towards a Happy You (HEALTHY) schools resource (HSR) is an online resource providing primary school teachers with guidance and resources to teach three health domains. These domains include Food and Health, Healthy Lifestyles and Physical Education, Physical Activity and Sport (PEPAS). In essence, the HSR was designed to remove the burden from teachers of finding appropriate teaching resources whilst supporting the delivery of specific health learning themes included within the CfE. Despite several primary schools in Scotland having trialled the resource, no formal evaluation has yet been undertaken. Since this resource is freely available to all schools within the Lanarkshire region of Scotland, there is potential for this resource to be used by hundreds of teachers when devising health and wellbeing lessons. Consequently, the potential impact upon children exposed to the teacher and their lessons may serve as a catalyst for improving PA and dietary behaviours.

It is essential that the efficacy of the HSR is established in order to inform future investment decisions in Scottish Education. A full examination of the effect of on the HSR would require a cluster randomized control trial (RCT). Cluster RCT’s are expensive to conduct and some RCT’s have been criticised as being ‘a black box’ since without adequate evaluation of the intervention process it can be difficult to know why (or why not) the intervention worked [23]. Therefore, before proceeding to a full trial it is important to; understand the intervention process and the active ingredients via which it works and to ensure that the intervention is feasible. It is also important to identify any aspects of the intervention which could be improved before progressing to a main trial and any unintended consequences resulting from HSR use. Indeed, without sufficient feasibility research and comprehensive process evaluations, the replicability and insights gained from future HSR evaluations will be very limited [24].

Therefore, this study aimed to evaluate the feasibility and preliminary effectiveness of the HSR for improving measures of health and wellbeing amongst Scottish primary school children. Recent recommendations regarding process evaluations suggest intervention outcomes should be captured alongside information regarding intervention implementation (training, reach, fidelity) and the mechanisms of impact using both quantitative and qualitative data [23,25]. In order to determine study feasibility of a teacher led HSR intervention therefore, we had five interlinking aims: 1) To assess the feasibility of recruiting and retaining primary school children and teachers to an HSR intervention; 2) To assess the feasibility of collecting outcome measures including markers of cardiometabolic risk and the suitability of the dietary knowledge questionnaire as a reliable and valid measure; 3) To examine potential changes in dietary knowledge, BMI, adiposity and cardiometabolic risk as an indication of the potential efficacy of the teacher led HSR intervention; 4) To conduct a process evaluation regarding teacher use of the HSR, intervention fidelity and the active ingredients which encouraged HSR use; and 5) To obtain teacher feedback to establish the appropriateness of the HSR, and possible improvements to both the HSR and the research process alongside any unintended HSR outcomes.

## Materials and methods

**Fig 1:**
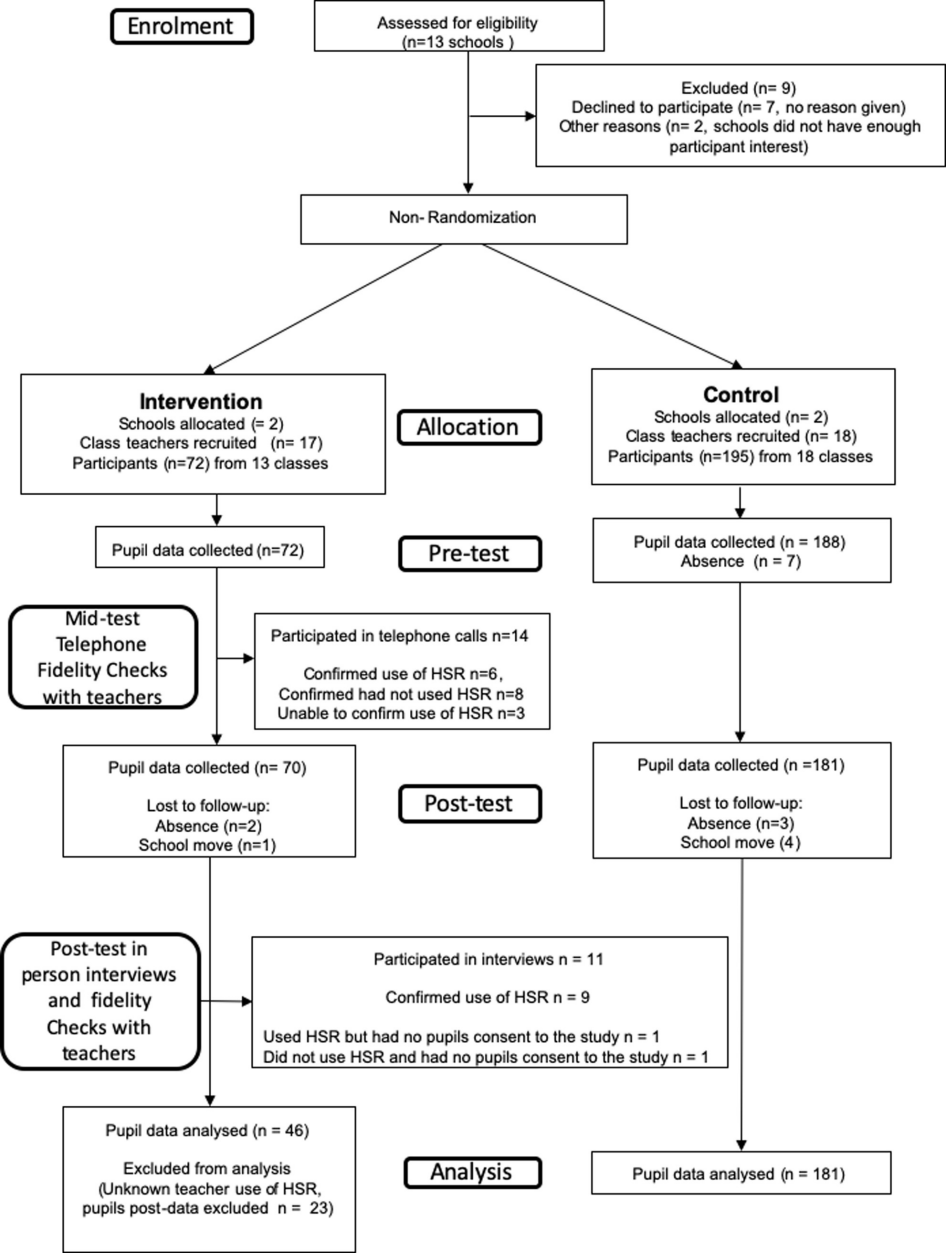
Consort flow diagram of the recruitment, adherence and analysis process.

This was a two-arm, parallel group, non-randomized feasibility study comparing the intervention group and usual practice (control) groups. The proposed methodology was granted ethical approval by the University of the West of Scotland Ethics committee (Approval # 4-8-15–001). Thirteen schools were contacted by telephone or email and meetings were arranged with interested Head Teachers to discuss the study proposal. The flow of participants can be found in Fig 1. Due to practical reasons surrounding staff availability and willingness to be randomized, we were unable to randomize schools to either the intervention or control arm of the study.

### Inclusion criteria

No inclusion or exclusion criteria were stipulated for the children other than all parents had to provide consent and complete a medical history questionnaire for their child’s participation in the study. From the medical history questionnaire, children were excluded if they suffered or had a history of any health issues that would prevent them from participating in the proposed study. From the returned medical history questionnaires, no child was excluded for any health issue. Due to minimal available resources and finances, a decision was taken to exclude schools who were unable to recruit a minimum of 20 participants. This decision was based on the premise that some participants would not consent to all outcome measures and there would be some drop-out at post-intervention.

### Intervention

The HSR was designed to support primary school classroom teachers to deliver the health and wellbeing experiences and outcomes outlined in the CfE across three key themes (Food and Health, Healthy Lifestyles and Physical Education, Physical Activity and Sport (PEPAS)). Intended to be cost and time efficient, the HSR can be accessed online (http://healthyschools.scot/) providing teachers with a curriculum based framework to guide the planning, tracking and monitoring of health and wellbeing education for each year group in the school. Specifically, the resource provides teachers with a tool that includes health and wellbeing learning criteria, suggested classroom activities and resources that can reduce their workload. In supporting the ethos of the CfE, the HSR is a non-prescriptive resource which emphasises flexibility of use, so that teachers can draw upon their professional competencies and adapt curriculum guidance to meet the needs of their learners. As part of the HSR progression framework, schools were encouraged to implement a monthly health focus across the entire school which was taught at differing levels based on age.

The HSR was made available to all teachers who agreed to participate in the study and use the resource. A formal training session lasting one hour was provided to these teachers by a member of NHS Lanarkshire covering how to access and navigate the HSR. Teachers were also afforded the opportunity to note any concerns or questions they had during this session and were then given a compliment slip featuring the HSR website address. Although the website was a public domain, at the time of data collection it had not been widely advertised to schools and was not easily accessed via google or other teaching sites. Furthermore, no teachers had access to the HSR prior to the commencement of the intervention.

Intervention fidelity relates to the extent to which the intervention was delivered as intended. Given the sensitivity of observing teacher practice within the classroom setting, it was not feasible to observe the use of the resource by the teachers and its impact on teaching directly. Nonetheless, we conducted teacher HSR use fidelity checks during the mid-point of the intervention via telephone interviews and face-to-face interviews post-intervention.

### Aims 1 to 3: Feasibility of recruiting teachers and children and collecting child outcome measures

Details of the number of teachers recruited and proportion of children for whom parental consent was obtained, both with and without blood sampling, were used to address aim one. In order to assess the feasibility of collecting outcome data (aim 2) and changes in outcome measures as a result of the intervention (aim 3) the following data was collected at baseline (week 0) and post-intervention (week 10–12).

Stature was measured barefoot to the nearest 0.1cm using a portable stadiometer (Seca Stadiometer, Seca Ltd, Birmingham, UK). Weight was measured barefoot with light clothing to the nearest 0.1kg on electronic scales (Seca Digital Scales, Seca Ltd, Birmingham, UK). BMI (kg/m^2^) was calculated using the equation; weight (kg) divided by stature (m) squared. Waist Circumference (WC) in cm was obtained using inelastic gulick tape in a standing position midway between the lower rib and the anterior superior iliac spine following a normal expiration [26]. Waist-height ratio (WHtR) was calculated by dividing WC (cm) by height (cm). To facilitate the analysis of results between different genders and ages, values of each variable were standardized using the following procedures. From measured stature and weight, participants were classified as obese/overweight, or a healthy weight using BMI-z scores relative to the UK 1990 BMI population reference data [27]. WC-z scores were calculated relative to the UK 1988 reference data [28] using software provided by the Child Growth Foundation [29].

Blood pressure (mmHg) was measured in an upright seated position using an automated sphygmomanometer (Omron M10-IT Blood Pressure Monitor HEM-7080IT-E, Omron Healthcare UK Ltd, Milton Keynes, UK) following a 10-minute rest period on the left upper arm. Blood pressure was converted to standardized z-scores using software provided by the Child Growth Foundation [29].

Blood samples were obtained using a finger prick method and analysed using the Cholestex LDX analyser (Cholestech Corporation, Hayward, California) [30,31]. Participants were required to fast for a minimum of 12-hours before sampling with breakfast provided immediately thereafter. Verbal confirmation of fasting was obtained prior to sampling. Blood samples were transferred into a cassette sample well and placed in the drawer of the analyser which provided a measure of triglycerides (TRGS), high density lipoprotein-cholesterol (HDL-c) and glucose (GLU). Skewed data were log-transformed where necessary and then transformed into age and gender specific z-scores. Finally, an age and sex adjusted continuous cardiometabolic risk (CMR) score (composite z-score) were calculated for each participant using the sum of the z scores of the following variables; GLU, TRGS, mean arterial pressure and inverted-HDL-c.

In the absence of a validated dietary knowledge tool which could be used across the age range of the study participants and was aligned with the HSR resource content, a dietary knowledge questionnaire (S1 File) was developed specifically for this study. As the questionnaire had not been previously validated, we took several steps to ensure its appropriateness for use in this study. The questionnaire was designed to reflect the CfE and content within HSR. Following its design, the questionnaire was reviewed by an experienced primary school teacher with expertise in delivering health and wellbeing lessons and the questionnaire was piloted on 5 pupils aged 6–9. The dietary knowledge questionnaire was developed to assess the dietary content covered within the resource to give an overall indication of a child’s current knowledge surrounding food and drink. The dietary knowledge questionnaire was composed of nine questions relating to the nature of a balanced diet, healthy and unhealthy foods, portion size, appropriate behaviour when eating and cooking, and the effects of an unhealthy diet. There were four multiple choice questions which required the child to choose the most appropriate picture (using a similar format as adopted by Turner [32]) and five open questions allowing a child to demonstrate a broad or narrow understanding of each concept (e.g., what do you need to eat for a balanced diet). All answers were scored using a points system based on a marking criteria with negative marking, so that one mark was given for a correctly circled picture or correct piece of information and one mark was deducted for an incorrectly circled picture or incorrect fact or response. The marks for each question were then summed to provide an overall score.

Participants were invited to complete the dietary knowledge questionnaire individually with a researcher present to aid with reading and writing. All participants were reassured that the questionnaire was not a test and would not be given to their teacher or parents. All questions were read out loud to the participants and all answers were written by a researcher on behalf of each participant. If a participant did not understand the question, the researcher would explain the question to aid understanding using standardised prompts and if the participant remained unclear the researcher moved on to the next question.

### Aim 4: Process evaluation

Given the sensitivity of observing teaching practice within the classroom, we were not able to evaluate how often teachers used the resource and its impact on teaching directly. A teacher log book could have been used to track HSR use, however they typically have very poor completion rates [33] and were not accepted by the school teachers recruited. Therefore, we conducted dose and fidelity checks of the teachers use of the HSR midway through the intervention via telephone interviews (S2 File) and post-intervention via face to face interviews (S3 File). Due to teacher availability, interviews with teachers were undertaken between weeks 10–15 after the intervention commenced. During these interviews we established whether teachers had used the HSR, which areas of HSR they used as well as frequency of use. To obtain further data regarding dose, we collected data regarding the frequency of teaching health and wellbeing by the classroom teachers before and after the intervention via a brief self-report questionnaire. The questionnaire was rated on an 8-point scale in response to the question ‘Approximately, how often do you teach health and wellbeing topics in class?’ (1 = less than once a month, 2 = once a month or more, 3 = once a fortnight, 4 = once a week, 5 = twice a week, 6 = 3 times a week, 7 = 4 times a week, 8 = everyday).

### Aim 5: Teacher feedback

Following the intervention teacher interviews were conducted by the second author to obtain further information on the following topics: 1) the active ingredients which facilitated teachers’ use of the HSR 2) the perceived outcomes of using the HSR for teachers and pupils, including any unintended outcomes and 3) recommendations to improve the HSR and teachers’ perceptions regarding research feasibility.

### Statistical analyses

#### Aims 1 to 4: Quantitative data

The primary outcome of this study was to understand the feasibility of implementing the HSR in a larger study. Therefore, the main statistical analysis performed was descriptive in nature with the main aim of recruiting and retaining sufficient participants as well as generating a preliminary estimate of effect from the intervention. Moreover, the study was not powered to detect significant between group differences and as there were insufficient clusters within the sample, we were unable to adjust for school clustering in the analysis. Previous recommendations suggest that between 24 and 50 participants are needed to justify a feasibility study [34,35]. However, these recommendations are not based on clustered designs and may be inappropriate for this study. The lack of population follow-up estimates for dietary knowledge, cardiometabolic risk indicators and measures of adiposity from studies utilizing the same design, intervention and population also precludes the use of previously published data to inform our sample size.

Nonetheless, the intervention and comparison groups were compared using univariate Analysis of Covariance (ANCOVA) for eleven dependant variable measures and the self-reported data of the frequency of teaching health and wellbeing. Post-intervention measures were used as the dependent variable, group as the independent variable, and the baseline measure as the covariate as recommended [36] to estimate preliminary effectiveness. The 95% confidence intervals (CI) for the mean differences for each group are presented with the partial eta squared values *(η*_*p*_ 2) reported as effect size estimates. In accordance with Green and colleagues [37], 0.01, 0.06 and 0.14 were interpreted as small, medium and large effect sizes, respectively. Only participants that had complete data (i.e. both baseline and post-intervention measures) were included in the ANCOVA for each variable.

To evaluate the suitability of the dietary knowledge questionnaire, an inter-rater reliability test was conducted whereby the third and last authors independently scored 50 completed questionnaires with scores subsequently compared using Intraclass Correlation. The scale’s reliability was assessed via Cronbach alpha coefficients as indications of internal consistency. Furthermore, the questionnaires predictive validity was assessed via correlations between pre-intervention scores of dietary knowledge and BMI-z, WC and WHtR at both pre- and post-intervention. All analyses were conducted using IBM SPSS Statistics 22 (IBM Chicago, IL, USA).

#### Aim 5: Qualitative data

All the interviews were audio recorded and transcribed with thematic analysis undertaken using a framework approach [38]. To generate familiarity with the data, the audio files from the interviews were listened to and the transcripts read by the second author. Initial themes were outlined deductively based upon the apriori study aims. Transcripts were then inductively analysed to identify all quotes which were deemed relevant to the research study regardless of the deductive themes to ensure no data of interest was overlooked [39]. All relevant quotations were then charted under the deductive themes within the framework whilst continually adjusting the thematic framework to add and edit themes to incorporate the data which had emerged inductively.

From the summaries, mapping took place where paper-based visual displays of themes, subthemes and example quotations were created and scrutinised before the findings were written up as thematic tables. Several steps were taken to ensure credibility and dependability of the data. Several iterative discussions took place whilst the team viewed the visual displays where the meaning of interpretations and themes were discussed and questioned. This process assisted the clarification of themes/subthemes and on several occasions the team returned to original transcripts to confirm a credible interpretation. Member checks were also offered to all 11 teacher participants to review and amend their individual transcript via email. Of these 11 teachers, two replied with one making minor spelling amendments to their transcript.

The qualitative element of the study was designed and reported in accordance with the Consolidated Criteria for Reporting Qualitative Research (COREQ) for interviews and focus groups [40].

## Results

### Aims 1 and 2: Recruitment and data collection

A total of 13 schools were contacted and six agreed to participate. Initial recruitment was challenging as seven of the schools were unwilling to participate as their teachers had already created annual class plans in advance of the school term and were unwilling to accommodate the study. After communicating this information to the resource developers, NHS Lanarkshire agreed to provide incentives to schools who were interested in participating in the study. These incentives consisted of £500 worth of vouchers for schools to purchase sport related equipment. Of the remaining six schools we contacted, all agreed to participate but due to factors related to staff availability and some schools wishing to focus more on health and wellbeing as part of their school improvement plans, only 3 schools were willing to be randomized. Also, two of the schools (one of which was willing to be randomized) who agreed to participate were unable to generate enough interest and consent for pupils to participate (*n* pupils < 20). Therefore, from the 13 schools who were approached to participate in the study, six (46%) agreed to participate although only four (31%) could generate sufficient interest and consent from study participants. A total of 35 classroom teachers agreed to participate in the study and the HSR was made available to 17 teachers assigned to the intervention group. In each school, recruitment ranged from 23 to 169 children who provided consent. The flow of participants throughout the study is shown in Fig 1. The recruitment, consent forms and provision rates provided at baseline and post-intervention are shown in Table 1 whereas the descriptive characteristics of the study sample at baseline and post-intervention are shown in Table 2.

**Table 1:** Recruitment, consent rate and data provision of the four participating schools.

School	Arm Allocation	No. of Consent forms delivered (*n*)	Signed Consent (*n*)	Consent with Blood Sampling	Provided Baseline Data (*n*)	Provided Post Measure Data (*n*)	Post data removed due to teacher fidelity (*n*)
1	I	47	23	20	23	21	6
2	C	60	26	25	25	24	-
3	I	220	49	39	49	48	17
4	C	287	169	117	164	158	-
Total	-	614	267	201	261	251	23

I = Intervention; C = Control; *n* = number of participants

**Table 2:** Descriptive characteristics for anthropometric and blood marker data of participants by arm group at baseline and post-measure.

Variable	Intervention		Control	
	Baseline Mean ± SD (*n*)	Post-Measure Mean ± SD (*n*)	Baseline Mean ± SD (*n*)	Post-Measure Mean ± SD (*n*)
BMI (kg/m^2^)	16.9 ± 2.6 (72)	17.2 ± 2.9 (69)	17.3 ± 3.1 (188)	17.45 ± 3.08 (181)
Height (cm)	126.9 ± 9.1 (72)	129.5 ± 8.4 (69)	130.0 ± 13.061 (188)	131.53 ± 13.64 (181)
Weight (kg)	27.5 ± 6.6 (72)	29.2 ± 7.5 (69)	29.9 ± 9.9 (188)	30.94 ± 10.23 (181)
Waist Circumference (cm)	59.2 ± 7.3 (72)	58.4 ± 8.4 (69)	60.7 ± 8.0 (188)	57.88 ± 8.80 (181)
WHtR	0.46 ± 0.0 (72)	0.45 ± 0.05 (69)	0.47 ± 0.05 (188)	0.44 ± 0.05 (181)
Systolic BP (mmHg)	112 ± 12 (72)	113 ± 11 (65)	107 ± 10 (185)	108.96 ± 12.21 (177)
Diastolic BP (mmHg)	70 ± 10 (72)	72 ± 13 (65)	66 ± 10 (185)	66.77 ± 12.22 (177)
MAP	84 ± 10 (72)	86 ± 11 (65)	80 ± 10 (185)	80.50 ± 11.23 (177)
HDL-c (mmol/l)	1.4 ± 0.3 (59)	1.4 ± 0.4 (47)	1.5 ± 0.3 (112)	1.48 ± 0.33 (86)
Triglycerides (mmol/l)	0.7 ± 0.3 (59)	0.7 ± 0.3 (47)	0.8 ± 0.4 (112)	0.77 ± 0.48 (86)
Glucose (mmol/l)	4.5 ± 0.5 (59)	5.0 ± 0.7 (47)	5.0 ± 0.5 (112)	5.18 ± 0.49 (86)
Cardiometabolic risk	-0.1 ± 2.5 (59)	0.07± 2.2 (46)	0.2 ± 2.0 (108)	0.01 ± 2.41 (84)
Total Dietary knowledge	11.9 ± 4.1 (69)	15.0 ± 4.1 (64)	12.9 ± 4.2 (188)	13.96 ± 3.82 (175)
Teaching of H&W	5.76 ± 1.56 (17)	5.56 ± 1.24 (12)	6.70 ± 1.25 (10)	6.75 ± 1.39 (8)

Values presented as mean ± (SD). BMI = Body mass index; WHtR = waist to height ratio; MAP = mean arterial pressure; HDL-c = high-density lipoprotein cholesterol; BP = Blood Pressure. The number of subjects (n) are provided for each outcome. H&W = Health and wellbeing.

Initial consent forms contained more information and questionnaires for parents to complete regarding their child’s current physical activity levels and quality of life. However, informal consultation with parents from a non-participating school suggested that the consent packs were lengthy and overly time-consuming to complete. Therefore, a revised pack containing only the required consent forms and information sheets were issued to the final two schools (schools 3 and 4) resulting in more parents providing consent for their child’s participation. It is important to highlight that the number of consent forms distributed to schools 1 and 2 differed to schools 3 and 4. Since the main aim of this study was to evaluate the feasibility of teachers to deliver the HSR to children within the classroom setting, we did not specify an age restriction for involvement. Nevertheless, in schools 1 and 2 it was agreed with the Head Teachers that only specific classes would be recruited in the first instance with the option of further recruitment later depending upon initial recruitment. In school 3, we decided to explore the rate of uptake when attempting to recruit participants from the whole school as requested by one of the Head Teachers. Since recruitment was low from school 3 (22% uptake), we decided to follow a similar strategy with school 4 with recruitment made available to the whole school cohort. Unexpectedly, the uptake from school 4 (59% uptake, *n* = 169) exceeded expectations. Consequently, there was a noticeable difference between the number of participants who consented to the control group (*n* = 195) compared to the intervention group (n = 72) which may in part be due to our initial recruitment strategy.

The mean consent form return across the four schools was 43%, of which 75% agreed to blood sampling. The drop-out rate between baseline and post-intervention data collection was low (6%) suggesting that the intervention was acceptable to participants and comparable with previous studies [41]. Despite providing consent for the blood sampling, we were unable to capture a significant number of samples from participants. We were unable to capture baseline values from 30 children for the following reasons: absent n = 5; non-fasted n = 5; refused sampling n = 8; sampling error n = 12. At post-intervention, we were unable to capture values from 56 children for the following reasons: absent n = 3; non-fasted n = 18; sampling error n = 12; school move n = 4; refused sampling n = 19.

The dietary knowledge questionnaire revealed excellent reliability with a high degree of agreement evident between the two raters with an average Intraclass Correlation = 0.99 (with a 95% confidence interval from 0.99 to 1.00) *F* 121.433 (49), *p* < 0.001. A good level of internal consistency was found with Cronbach alpha coefficients of 0.72 (pre-intervention) and 0.71 (post-intervention). Findings also suggest that the questionnaire has some predictive validity as pre-intervention scores on the dietary knowledge questionnaire (Z-scored) were significantly negatively correlated with WHtR at both pre- (*r* = -0.31, *p* > 0.001) and post-intervention (*r* = -0.30, *p >* 0.001). This suggests that lower levels of dietary knowledge were related to a higher WHtR.

### Aim 3: Changes in outcome measures

Table 3 shows the differences found between the control group and the intervention group with between group differences always compared to the control group. BMI-z, Glucose-z and dietary knowledge showed improvements when compared to the control group. Nonetheless, the effect size for each outcome was small (≤0.06).

**Table 3:** Changes in outcome measures from pre-to post-intervention in the control and intervention groups.

Variable	Adjusted C-I Difference (95% CI) *	Effect size Partial eta squared
BMI-z	0.07 (-0.19, 0.05)	0.01
Waist Circumference-z	-0.33 (-0.63, -0.04)	0.02
WHtR	-0.15 (-0.38, 0.08)	0.01
Systolic BP-z	-0.39 (-0.72, -0.06)	0.03
Diastolic BP-z	-0.44 (-0.77, -0.11)	0.03
MAP-z	-0.44 (-0.77, -0.11)	0.03
HDL-C-z	0.08 (-0.20, 0.36)	0.00
Triglycerides-z	-0.07 (-0.30, 0.43)	0.00
Glucose-z	0.05 (-0.40, 0.50)	0.00
Cardiometabolic risk	-0.72 (-1.58, 0.14)	0.03
Dietary knowledge	-1.42 (-2.20, -0.63)	0.06
Teaching of H&W	0.82 (-2.10, 0.44)	0.10

*Differences in mean change and 95% confidence intervals (CI) are the differences between the control and intervention groups after adjustment for baseline values of the outcome measure. Between group differences always compared to the control group. Analysis not clustered for school due to insufficient clusters.

### Aim 4: Intervention fidelity & HSR use

Efforts to have teachers quantify how much they used the resource when delivering lessons proved challenging, with log books and observations not welcomed by teachers.

Of the 13 teachers who taught participants within their class and provided consent, 3 failed to participate in mid-intervention fidelity checks and 1 failed to participate in post-intervention fidelity checks. As such, an additional 23 pupils were removed post-intervention as we were unable to confirm whether they used the resource. All nine teachers who completed the post-intervention fidelity checks confirmed they had used the HSR, and that health and wellbeing lessons were delivered to the children using materials taken from the HSR. The additional teacher interviewed who had not used the HSR suggested that an earlier introduction of the HSR prior to term planning would have made them more likely to use the resource.

Although the interview guide included a question about how often the teachers had used the HSR, many teachers gave vague answers which were difficult to quantify. Furthermore, there were no significant differences pre- and post-intervention in the self-reported teaching of health and wellbeing as a result of using the HSR (See Table 3). The curriculum area of Food & Health was reported to be the most regularly used and applied within classroom teaching with some teachers stating that they were most confident teaching topics related to food in comparison to topics covering Physical activity, Physical Education and Sport, and Healthy Lifestyles. Indeed, when asked about their use of HSR, physical activity did not seem to be a focused topic.

### The active ingredients which facilitated teachers’ use of the HSR

There were several factors which encouraged teacher use of the HSR (see Table A in S4 File). The HSR was described by teachers as having a systematic structure which was simple and progressive that provided curriculum relevant guidance. Teachers also discussed how they found the HSR accessible and easy to navigate with clear tabs and colour-coding. Alongside the relevance and ease of use of the HSR, the school context facilitated its use. The teachers discussed the school management team and the school health and wellbeing coordinator as positively influencing their use of the HSR. Indeed, promotion of the HSR by the management team and a whole school approach using the yearly planner where each class covered similar topics at the same time encouraged teachers to use the resource. The school’s health and wellbeing coordinator also supported teacher use of the HSR. In particular, some evening development sessions for teachers (commonly known as Collective Activity Time; CAT) were focussed on the HSR which were thought to be of value to the teachers using the resource for the first time.

### Aim 5: Post intervention teacher feedback

Teachers provided feedback concerning: 1) the perceived outcomes of using HSR for teachers and pupils, including any unintended outcomes 2) difficulties and recommendations to improve the HSR, and 3) perceptions of research feasibility (See Tables A-C in S4 File for an overview of themes and example quotations). In addition to the nine teachers who had participants within their class consent to the study aims, an additional two teachers who had used the HSR but did not have any participants consent to the study aims, agreed to be interviewed. Of these two additional teachers, one teacher acknowledged that they had never used the HSR. Thus, interviews lasting 23.1 ± 8.1 minutes were conducted with 11 teachers within their respective school.

### The perceived outcomes of using HSR for teachers and pupils

Teachers discussed how the HSR provided effective guidance and saved them planning time. Using the HSR made them more enthusiastic about teaching health and wellbeing and encouraged them to deliver more varied health and wellbeing lessons. Indeed, teachers reported teaching health and wellbeing more because of the HSR. Teachers felt that the pupils’ needs were being met through the HSR and that pupils were talking more about health and wellbeing than they did before having been exposed to the HSR. The teachers also suggested that the HSR monthly focus created a sense of community in school and helped to embed the children’s knowledge. The monthly focus also kept their teaching on target and teachers noted they were less likely to skip health and wellbeing lessons. Teachers also discussed an unintended outcome of participating in the research study stating that they reflected more on their practice, what they are teaching regarding health and wellbeing and how they are teaching.

### Recommendations to improve the HSR

Teachers were asked to describe any difficulties and provide recommendations to improve the HSR. Despite many teachers commenting on the ease of using the resource, some teachers highlighted minor issues with functionality of the website (See Table C in S4 File). In particular teachers could not locate the website easily via google. Whilst this was intentional given potential contamination effects and the need to prevent teachers in the control arm from accessing the HSR, this also hindered access for intervention teachers. One teacher who failed to use the HSR suggested introductory HSR training could have been more extensive. In terms of HSR tools and features, some teachers suggested they would have liked standardised templates to use, encouraging consistency of teaching across schools. Finally, teachers suggested that the HSR could feature more assistance regarding assessing and tracking the progress of pupils within health and wellbeing.

### Teachers’ perceptions regarding research feasibility

In preparation for a larger-scale research study of the HSR, teachers were asked to discuss the feasibility and potential improvements to the research process. Teachers discussed the time commitment positively as they did not have to give up any planning time or personal time to take part in the study and did not receive too many emails. The telephone call fidelity interviews and face-to-face interviews conducted in school during class time made it convenient for the teachers taking part. Teachers found the interview questions to be appropriate and found the study to be helpful in providing them with a useful resource. However, some teachers discussed how they would be more inclined to use the HSR had it been introduced at the beginning of the academic year before they had completed their lesson planning.

Within the current study, participant recruitment was 43% and teachers suggested several strategies which could have increased recruitment. All schools were offered the opportunity for information sessions and visits from researchers (which were not always taken), however, the teachers suggested offering parents more opportunities to gain information about the study could have increased recruitment. Providing parents information regarding the study background and the testing involved via parents’ evenings, teacher provided talks and demonstrations could have increased recruitment. Teachers also suggested that the use of rewards and posters could have encouraged pupils, which may lead to increased popularity with parents.

## Discussion

Findings suggest that the HSR is a feasible teacher-led intervention which has the potential to create positive health outcomes for children. We now discuss the specific findings in relation to the five interlinking aims: 1) The feasibility of recruiting and retaining children and teachers; 2) The feasibility of collecting outcome measures; 3) The potential efficacy of the teacher led HSR intervention, 4) The intervention process including teacher use of the HSR, intervention fidelity and the active ingredients which encouraged HSR use; and 5) The appropriateness of the HSR, possible improvements to both the HSR and the research process, and any unintended HSR outcomes.

### Recruitment and data collection feasibility

In relation to the feasibility of recruitment and data collection we successfully ran the intervention in two schools with a further two schools acting as control groups using parental opt-in consent. Recruitment of schools to the study (4/13, 31% uptake) was lower than another pilot investigation designed to increase PA levels and improve dietary behaviours in primary school children (71% uptake) [42]. At baseline, 201 children (75%) provided consent for blood sampling suggesting that many parents were comfortable with providing consent for this measure. Retention of participants to the study was high (94%) with 66% of consenting children also providing post-intervention data for the blood sampling suggesting that the intervention was acceptable to participants and comparable with previous studies [41]. The dietary knowledge questionnaire seemed to be a reliable method of measuring the children’s knowledge of healthy eating and a balanced diet with good levels of internal consistency and excellent inter-rater reliability. The questionnaire also demonstrated predictive validity as the scores of dietary knowledge were negatively correlated with WHtR, suggesting that less knowledge was related to a larger WHtR. Furthermore, teachers reported their participation to be manageable and the HSR to be an effective teaching tool, which had a positive impact on the children they taught. Despite our findings suggesting that it was feasible to recruit and retain participants to this study, several amendments to the study protocol will be required prior to further work.

### Enhancing participant recruitment

There was less than 50% uptake from schools that were recruited. Although the process of recruiting schools began 3–4 months prior to the anticipated baseline data collection sessions, future investigations will need to ensure that schools are contacted prior to the start of the school term when teachers create their annual class plans.

Reflecting upon the differing rates of uptake between schools 3 and 4, it was evident that subtle differences between the schools may have accounted for the differing recruitment rates. For instance, school 4 provided regular updates to parents about all aspects of the school via online notice boards whereas school 3 relied upon sending letters to parents through their child informing them of the latest developments and initiatives concerning the school. Based on these findings, in future investigations we will endeavour to communicate information relating to study aims and objectives, measures and data collection times through the schools’ website to establish whether this mode of communication has a positive effect upon study recruitment rates. We will remove additional measures which rely on parents to complete on behalf of their child to reduce the burden of providing consent for their child’s participation in future studies. Furthermore, based on the teacher’s recommendations, we would encourage schools to host more opportunities for parents to attend evening meetings, demonstrations and posters to provide more information and exposure regarding the study to parents and pupils.

### Preliminary effectiveness of the HSR intervention

The findings in relation to fidelity suggest that all teachers who used the HSR confirmed that health and wellbeing topics were delivered regularly to the children using materials taken from the HSR resource. This is an important finding suggesting that it was feasible for teachers and schools to utilise the resource to deliver health and wellbeing topics within class. Moreover, post-intervention teacher interviews suggested that the HSR provided clear and effective curriculum relevant guidance, saved planning time, and encouraged the teaching of health and wellbeing more regularly with more varied content. Nonetheless, capturing the consistency of delivery between teachers and the volume of resources utilised was difficult to quantify as teachers were reluctant to complete daily log books nor was it feasible to observe teacher delivery.

Teachers also provided vague answers when asked specifically how often they used the resource during the post-intervention interviews which proved difficult to quantify. In light off this, and as others have documented [43,44], we reported the frequency of health and wellbeing lessons being taught before and after the intervention period. It was evident that the control group appeared to deliver more health and wellbeing lessons both at the start and end of the intervention period in comparison to the intervention group although no significant differences over time were evident between the groups. We acknowledge however that recall bias may have influenced these results [45] although given our non-randomized design, it could equally be that schools using the HSR were those that needed to improve this area of curriculum delivery. As implementation is a complex issue, direct observations during lessons may have provided more insight into the process of intervention implementation and help identify the differences in health and wellbeing content being delivered between the groups which could help explain intervention effectiveness. Although this was not feasible in this study, it has also been suggested that observations may interfere with the dissemination process by altering teachers normal behaviour [45] which highlights the challenges for researchers capturing the extent of intervention implementation. The use of a single measure that combines different process measures as used previously [45] may serve as an appropriate tool to gauge the extent of implementation in future studies once the tool has been validated.

It is important to consider the recommendations of others who suggest that the integration of key health and wellbeing components into the curriculum, collaboration between key agencies and leadership from school management are likely to be important for schools adopting health and wellbeing initiatives [19,20,46]. Rather than trying to establish a simple and time efficient method of recording HSR use and specific content accessed, perhaps more effort is needed to promote the resource and have “buy-in” from schools and class teachers as to the merits of the resource and how useful it can be in assisting with the planning and delivery of health and wellbeing content within the curriculum. One such finding from the interviews with teachers suggested that the HSR created positive outcomes for their pupils who were discussing health topics more frequently. This is something that teachers would find pertinent to them which could easily be quantified by them in the classroom setting.

Although we acknowledge that this feasibility study is not appropriately powered to make inferences, we did undertake a statistical analysis to gain an insight into the preliminary effectiveness of the intervention on the quantitative outcome measures. The analysis seemed to reinforce teachers’ suggestions since a greater post-intervention dietary knowledge was demonstrated by the intervention group in comparison to the control group, after controlling for pre-intervention levels. It has been suggested that children may benefit from having greater dietary knowledge that allows them to make informed choices about energy sources and total intake which could decrease the likelihood of becoming overweight or obese [47]. Nonetheless, owing to the small number of clusters, which could not be considered within the analysis, results should be viewed with caution. Further work is needed to confirm this finding.

More positive intervention effects upon measures of health and wellbeing of the participants were not anticipated given the short-term nature of the intervention and the teachers’ primary focus on diet, rather than multi-component teaching of diet, physical activity and lifestyle. Taken together, our findings may speculatively suggest that the use of the HSR resource as a teaching aid may assist teachers in the delivery of the food and health theme as recommended within the CfE, however amendments to the HSR and teacher use of the HSR could enhance effectiveness related to Food and Health, Healthy Lifestyles and PEPAS.

### Enhancing teacher use and the effectiveness of the HSR

The fidelity checks suggested that the HSR was used by teachers, and teacher interviews highlighted specific factors and active ingredients to HSR use which should be considered in future research. Our findings suggest that teachers could have been given additional support to use the HSR through more initial training. Moreover, forming close links between the school’s management team and health and wellbeing coordinator who are likely to further support and enhance teachers’ use of HSR seems prudent. The HSR use is also likely to be increased by facilitating the copying, pasting and editing content to use directly in teaching, locating the website easily via google and the inclusion of a search bar. This feedback has already been relayed to the NHS Lanarkshire HSR team and these amendments have now been made. Some teachers would have liked standardised templates to use to encourage consistency of teaching across schools and more assistance regarding assessing and tracking the progress of pupils within health and wellbeing, which have also been considered by the NHS Lanarkshire HSR team. Finally, further discussions are warranted with schools and teachers regarding classroom teachers’ role in facilitating physical activity, in order encourage greater utilisation of the HSR content.

It was encouraging to note the extent of health and wellbeing content being delivered by all teachers throughout the study period (Table 2). Typically, this ranged from 2–3 times per week which is comparable to other school based health interventions [48] but considerably greater than a recent clustered randomized control trial which focussed on educational approaches to improve measures of health and wellbeing [15]. In this study, Kipping and colleagues provided intervention schools with 16 lesson plans and teaching materials alongside 10 parent-child interactive homework activities and written information for parents on how to encourage healthy eating and activity behaviours over a 10-month period. Despite the intervention being ineffective at increasing activity levels, decreasing sedentary behaviour or increasing healthy eating, the authors contend that their intervention may not have been intense enough to improve these behaviours. From our findings it appears that teachers in Scotland do teach health and wellbeing regularly, the challenge now is to ensure that the information being delivered is appropriate and intense enough [15] to induce improvements in measures of health and wellbeing.

### Comparison with other studies

Since the main aim of this study was to examine the feasibility of recruiting and retaining primary school children and teachers to a HSR intervention, directly comparing our findings to that of others is challenging. We are nonetheless able to draw upon the main findings of other process evaluations of school-based health interventions [48–50]. The ability to recruit 31% of the schools initially contacted to participate in this study is broadly similar to the rates reported elsewhere [15,51]. For instance, Kipping and colleagues reported a recruitment rate of 40% (60 schools recruited from 148 schools invited) [15] whereas Adab and colleagues reported a recruitment rate of 27% (54 schools recruited from 200 schools invited) [51]. Although the number of schools we initially contacted were much smaller than in these studies, it is encouraging to note the comparable recruitment rate of schools to UK school-based health interventions which may help inform future evaluations of the HSR resource.

Time constraints are commonly listed as a problem for the successful implementation of school-based health initiatives [48,50] but this didn’t appear to be a barrier in this study. A strength of the HSR is that it has been developed to save teachers time in finding resources which support the delivery of specific health and wellbeing learning themes within the curriculum. A common theme from other process evaluations of school-based health interventions is the need to incorporate interventions within schools by modifying the curriculum and for school management to provide support to teachers [48–50]. This often comes with additional costs to free up teachers or support staff to implement the intervention as well as on-going support to ensure the continued delivery of the intervention either through training or the availability of additional resources from the intervention developers. All of which requires funding resources which may limit the long-term implementation of such school-based health interventions.

One of the main challenges to continued implementation is that school-based health interventions are often viewed by schools and teachers as yet another ‘add-on’ in what is already a very busy school life [19,52]. The need for a holistic, school-based health resource that aligns and integrates with the school curriculum was at the forefront of the development of the HSR. This is in contrast to previous school-based health interventions that may be described as ‘add-on’ where activities are applied on top of the school curriculum requiring extra classroom teaching and support from school management [52]. An important finding from the teachers in this study was the need for additional training prior to the commencement of using the HSR. As highlighted by others, it is essential that teachers have adequate training for the successful implementation and sustainability of curriculum based health interventions [52]. As such, we recommend that developing a more comprehensive training programme for teachers, which is developed in line with the school curriculum, be at the forefront of future developments by NHS Lanarkshire and local education authorities when refining the HSR.

### Strengths and limitations

There are several strengths associated with this study. Firstly, the recruitment and retention rates suggest that the participants, parents and teachers found being involved in the study acceptable and did not disapprove of the aims of the study. Moreover, a 75% uptake for the blood sampling suggests that many parents and participants were not concerned with the inclusion of this measure. A further strength of this study was the acceptability of the resource by teachers. In contrast to many other school-based lifestyle intervention approaches, the HSR has been developed to integrate and embed healthy lifestyle, healthy eating and active lifestyle learning resources into curricular health and wellbeing education every day. Since the resource is utilised by teachers to inform the delivery of daily health and wellbeing learning themes within the curriculum, the resource has a high degree of sustainability.

Limitations of this study include the small sample size, the short intervention period and the lack of randomisation. Yet, it must be acknowledged that this feasibility study was not intended to be adequately powered to make statistical inferences nor was it intended to randomise groups. The purpose of this study was to enhance knowledge relating to the feasibility and acceptability of the HSR, recruitment strategies, outcome measures and participant retention rates. Thus, several revisions are recommended prior to future studies which are provided below.

### Recommendations

Findings from this feasibility study have resulted in several recommendations. These include:

- Initial school contact should be made before the school term begins which may allow schools to incorporate the delivery of future intervention studies within their curriculum planning sessions.
- Parental information packs should remain simple with one informative letter, one medical history form and one consent form to avoid confusion and lack of participation.
- Discussions should take place with Head Teachers to explore the use of the school website to disseminate study information.
- Future evaluation of the HSR should provide more extensive introductory training for teachers and seek to form a closer link with the school management team regarding the use of the HSR within the curriculum.
- Further evaluation is needed to more accurately comment upon the reach, implementation and satisfaction of both teachers and pupils in the use of the HSR to teach health and wellbeing lessons within the school curriculum.
- Finally, evidence suggests that children exposed to school-based PA interventions are nearly three times more likely to engage in health enhancing levels of PA. Thus, future work should consider introducing a PA intervention in conjunction to the HSR resource.

## Conclusion

In this study, we have demonstrated that it is feasible to recruit participants to participate in a school-based intervention designed to facilitate the teaching of health and wellbeing and improve such measures in children. Our findings suggest that use of the HSR has the potential to positively influence the dietary knowledge of children. Furthermore, teachers who used the HSR reported their participation to be manageable and the HSR to be an effective teaching tool that had a positive impact on the children they taught. These initial findings provide promising evidence that with some refinements, a greater sample size and a longer intervention exposure period, this school-based health intervention may result in improvements in the health and wellbeing of primary school children.

## Supporting information

S1 File Diet questionnaire. (DOC)

S2 File Teacher telephone fidelity check interview guide. (DOCX)

S3 File Teacher post-intervention interview guide. (DOCX)

S4 File Qualitative data. (DOCX)

S5 File Teacher data. (SAV)

S6 File Pupil data. (SAV)
