# Supplementary material for: A feasibility study with process evaluation of a teacher led resource to improve measures of child health
Source: PLoS One. 2019 Jul 2;14(7):e0218243. doi: 10.1371/journal.pone.0218243 (PMC6605653; doi:10.1371/journal.pone.0218243)

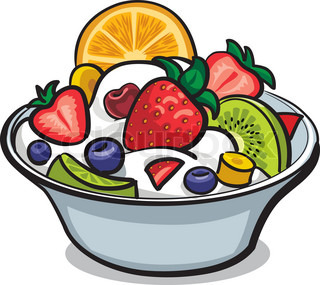


Diet Questionnaire

Name: _________________________

Age: _________________________

1. What do you need to eat for a balanced diet?
2. What should you do before cooking?
3. How should you act when at the table with your family or friends?

1. What could happen to your health if you have a poor diet?
2. Identify foods which are healthy and foods which are less healthy

Circle the healthy foods and cross out (X) the less healthy foods.

**
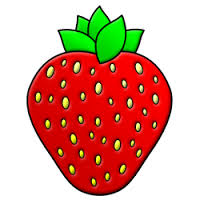

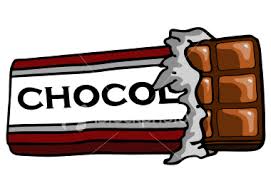
**
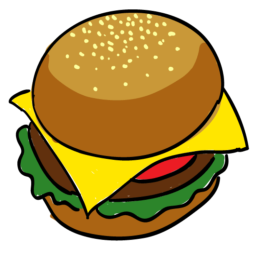
 **
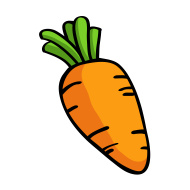
**


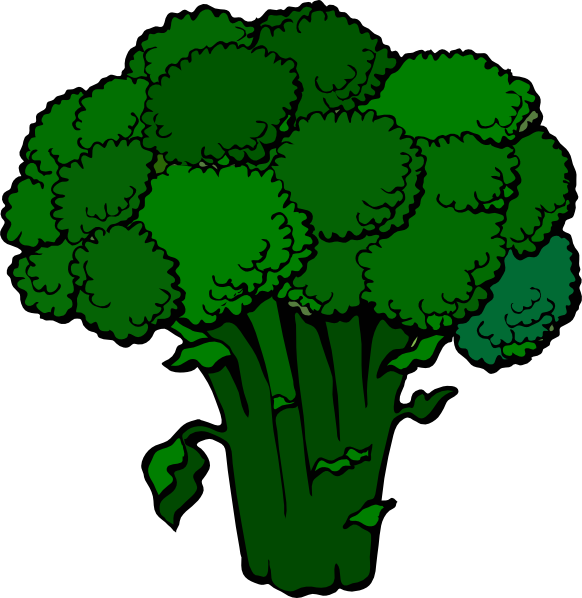

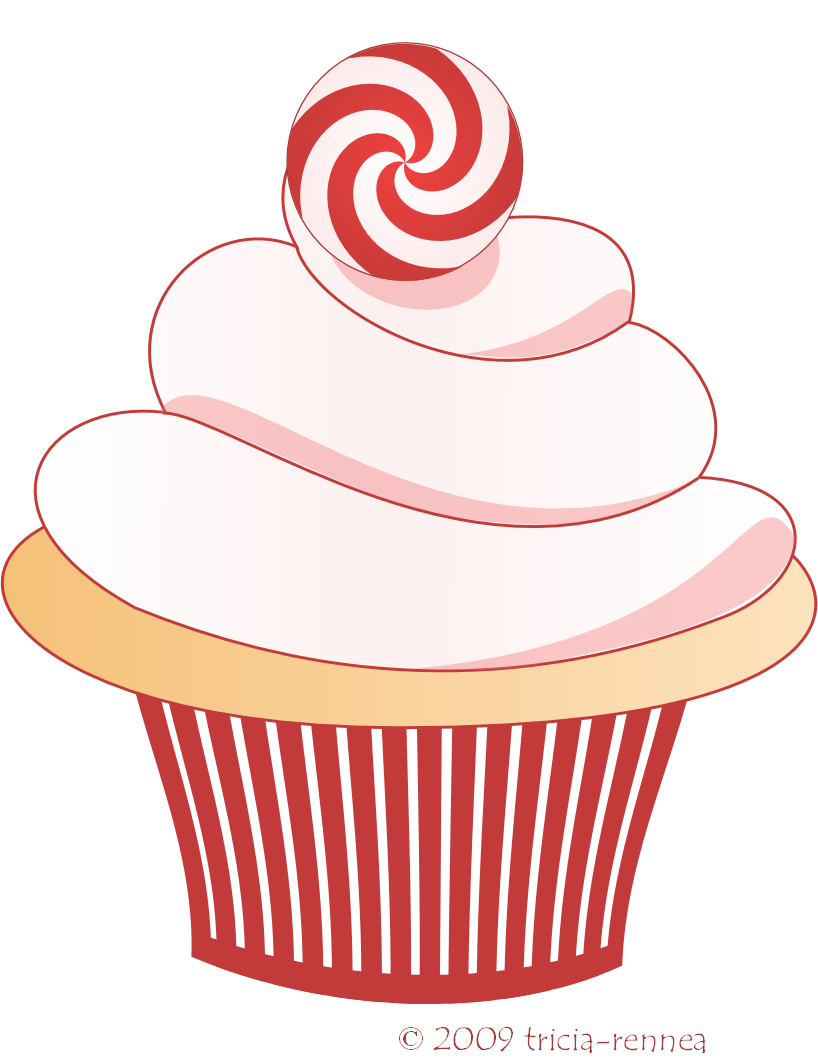

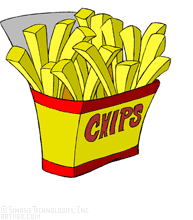

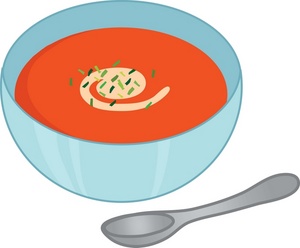


1. Circle what you think would be part o f a healthy breakfast?


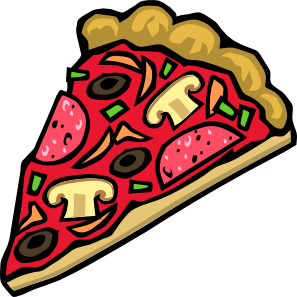

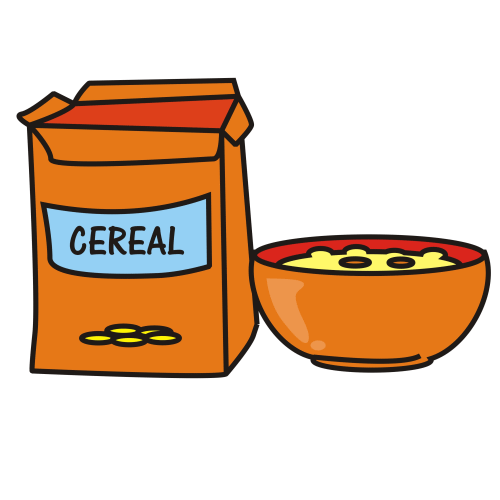

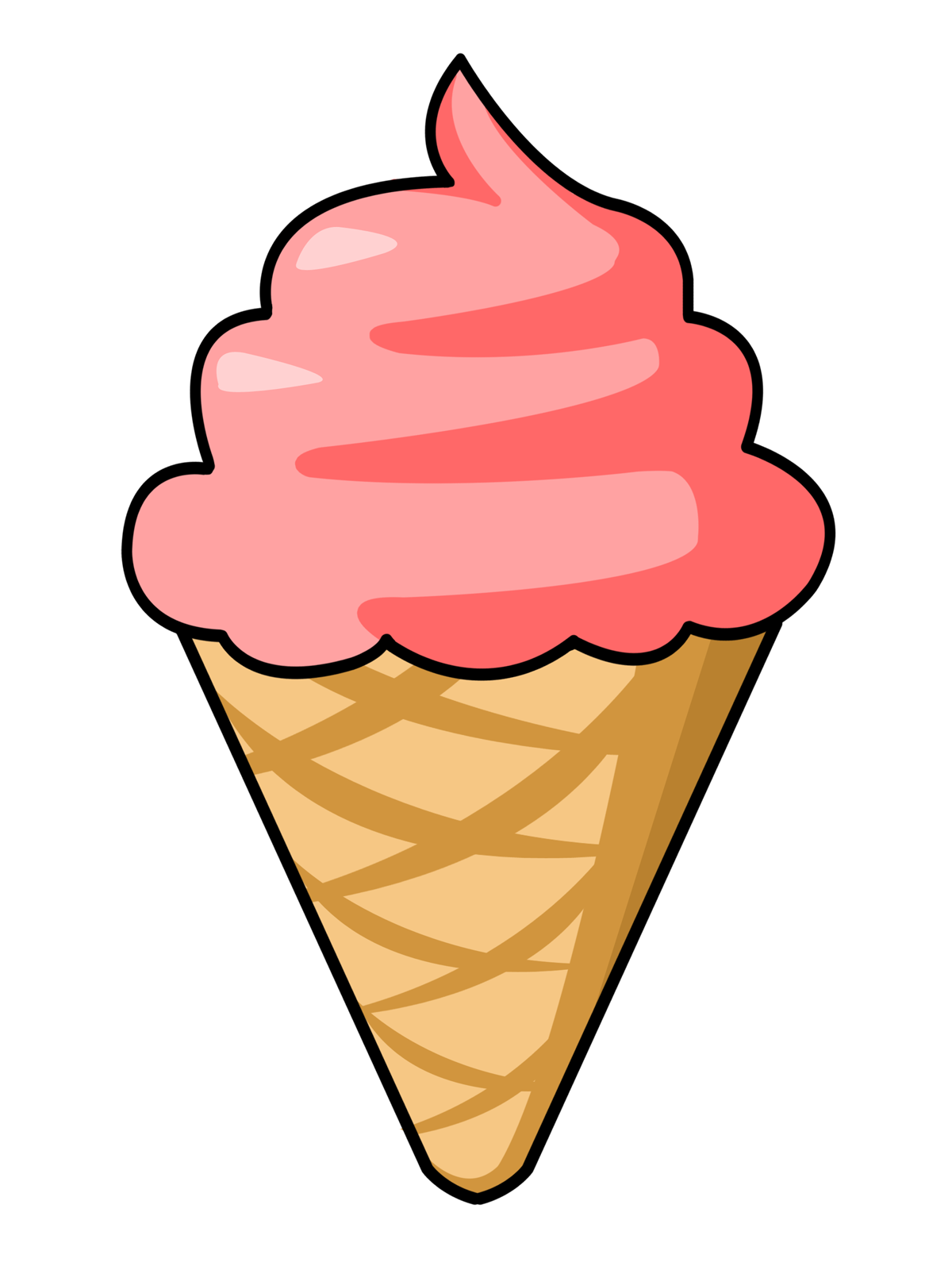

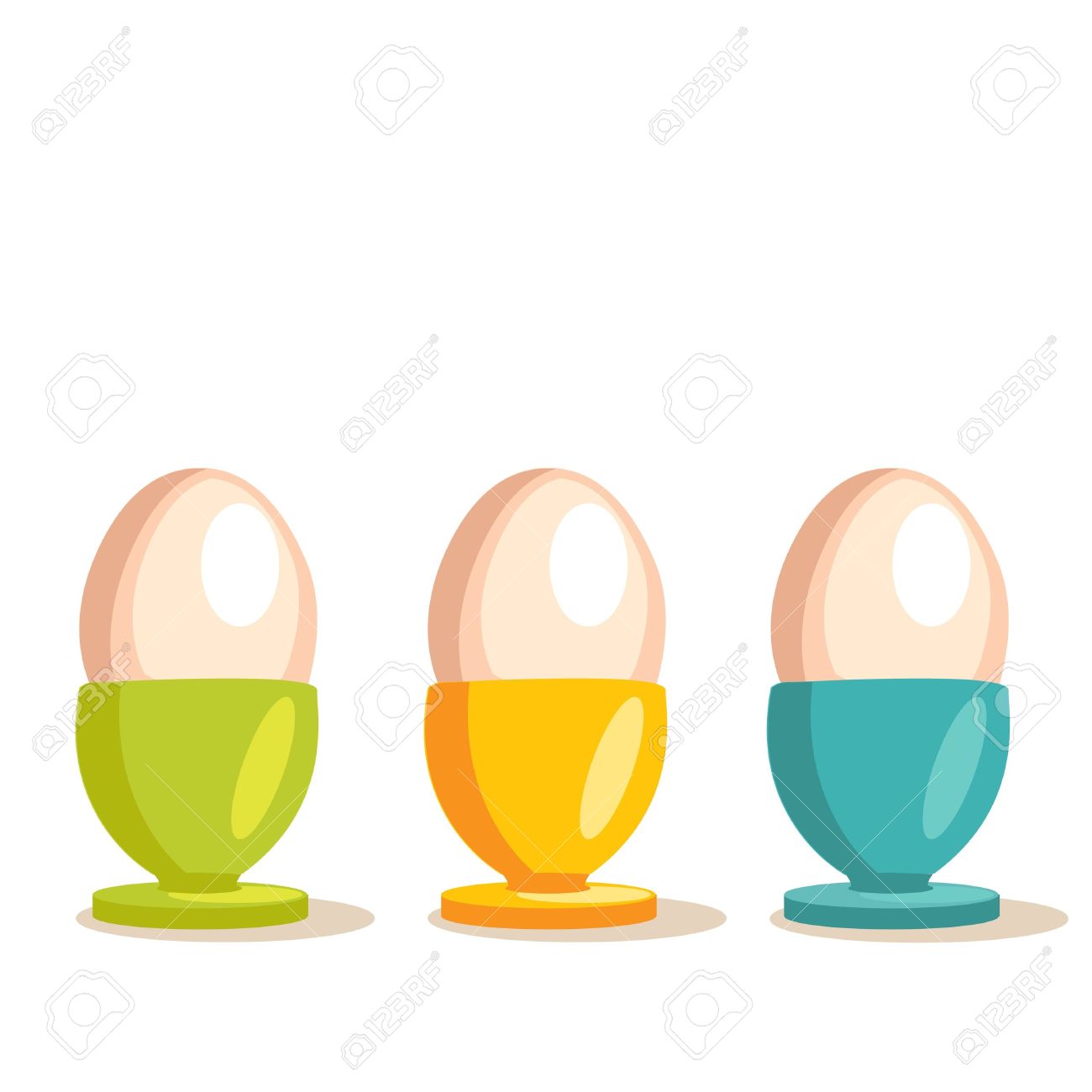


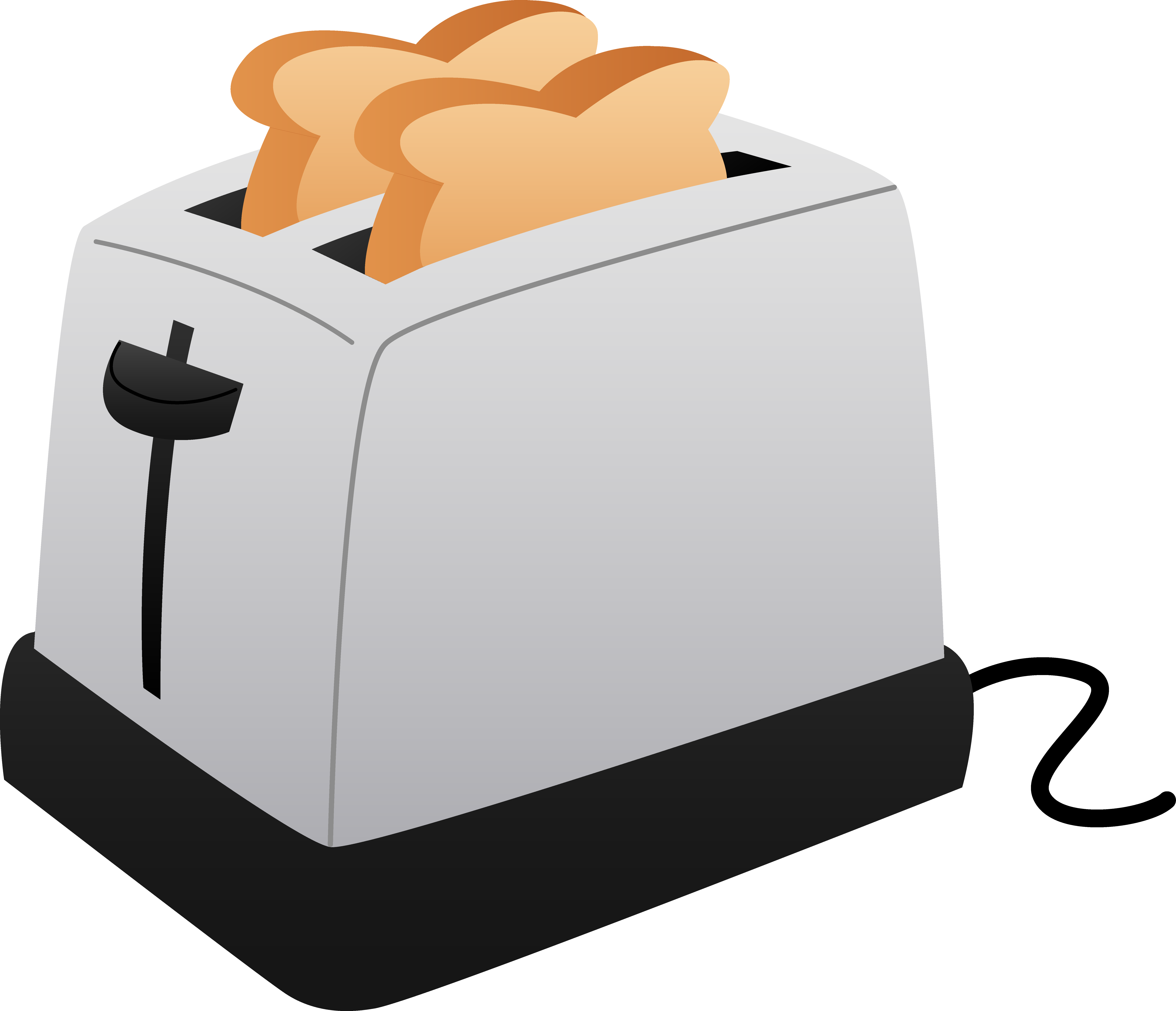

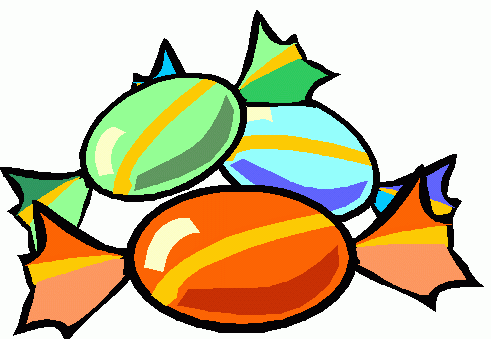

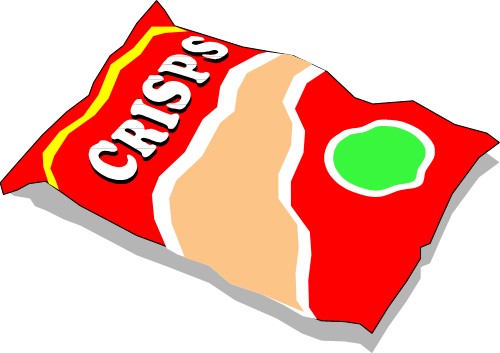

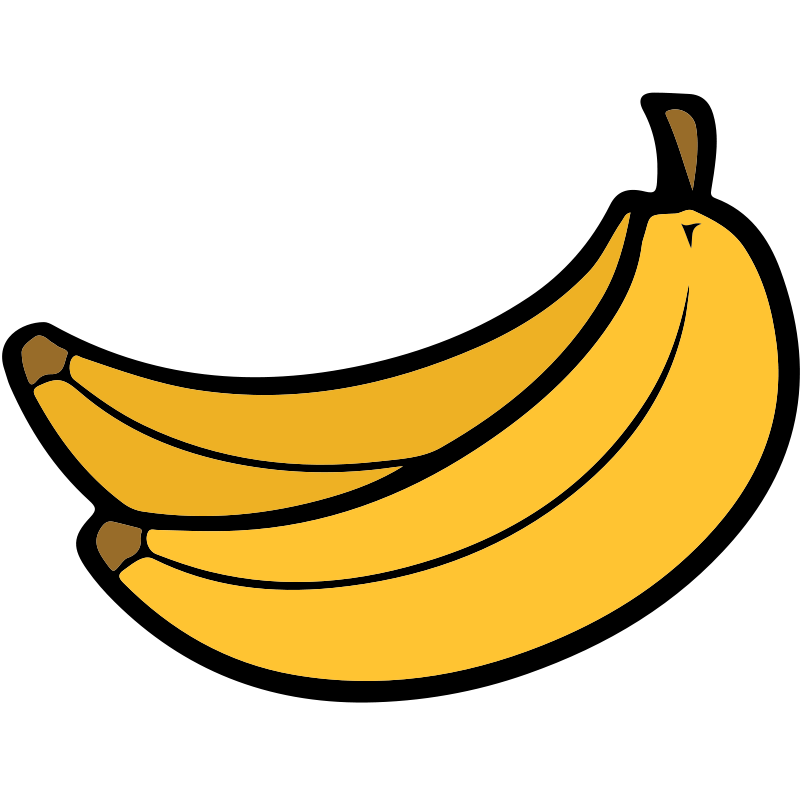


1. In what ways will eating a healthy breakfast help you?
2. What do you think is most healthy to drink? Circle your answer(s).


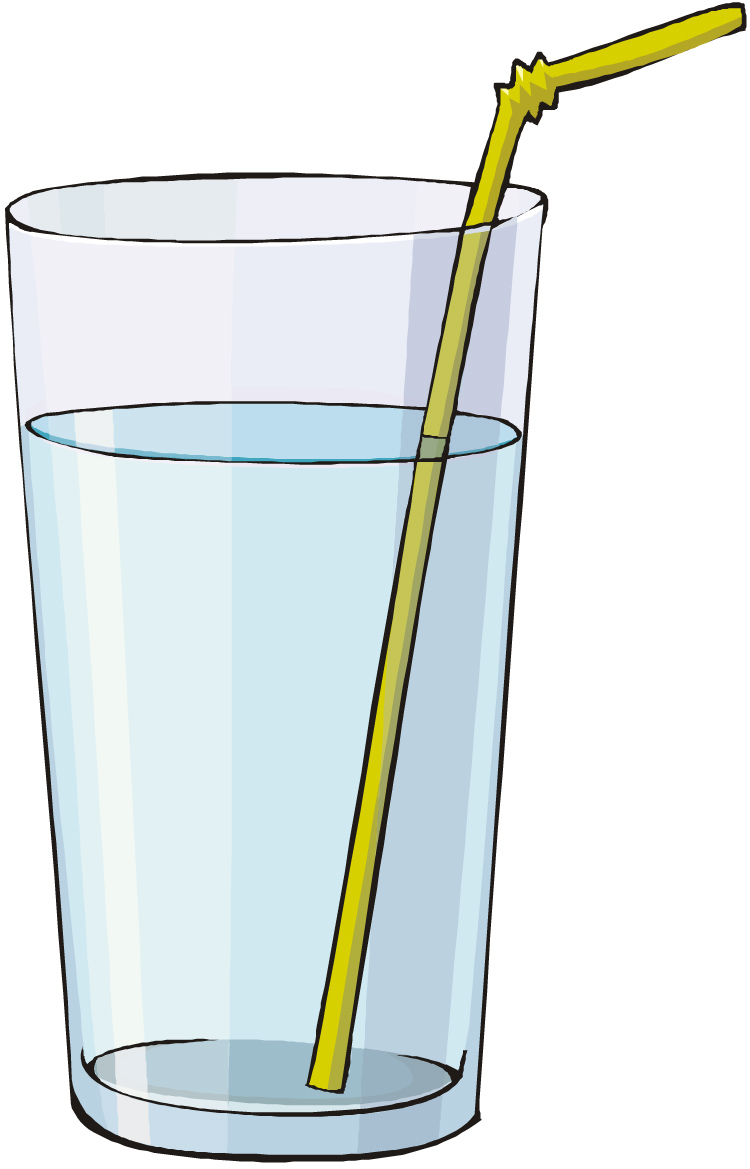

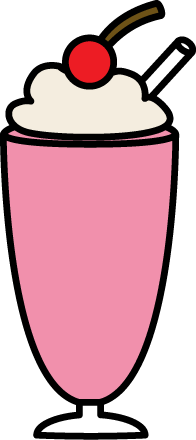

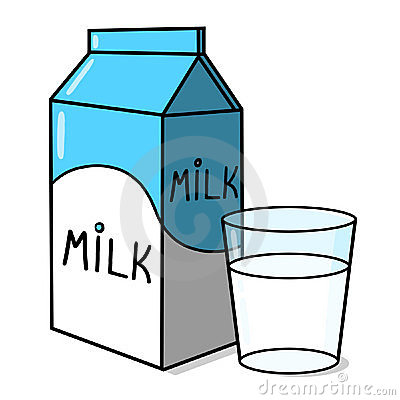

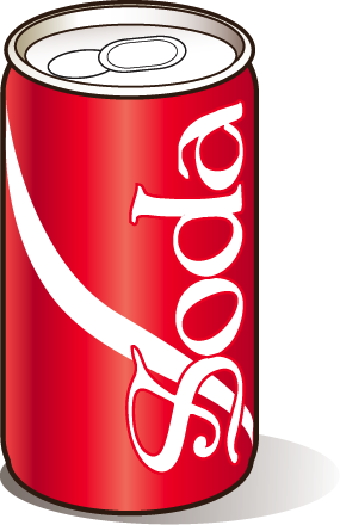


1. Chocolate can be part of a balanced diet. As a treat, how much chocolate do you think is healthy for you to eat? Circle your answer.


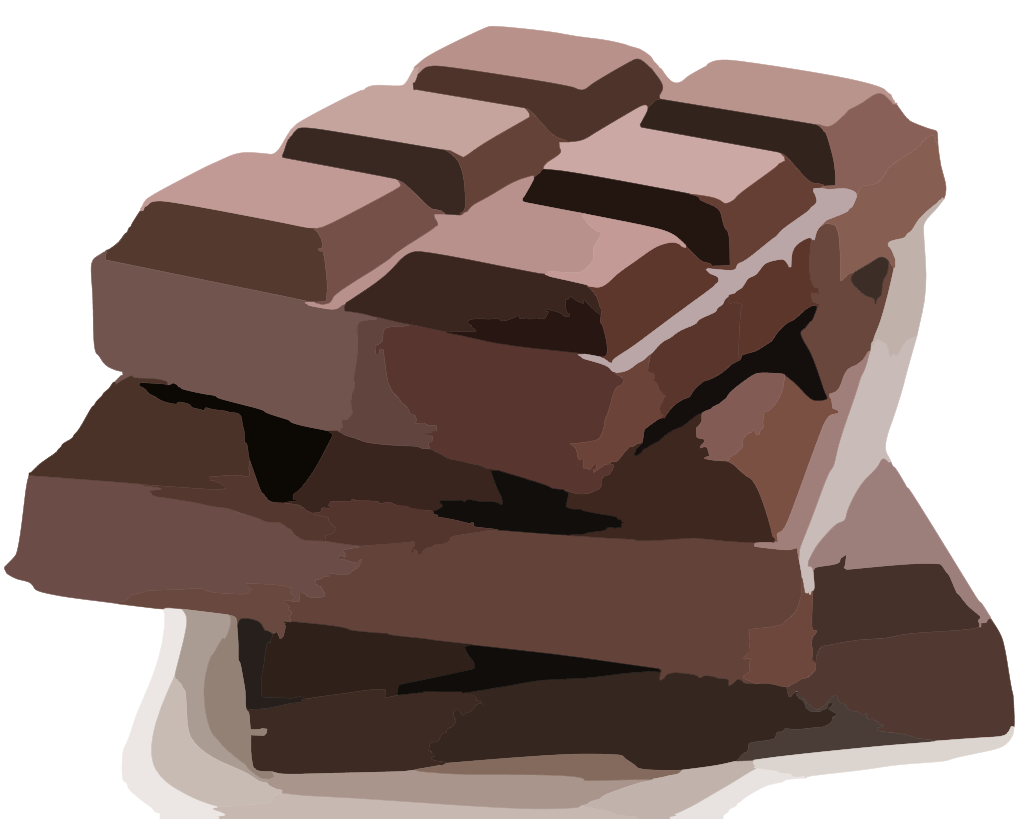

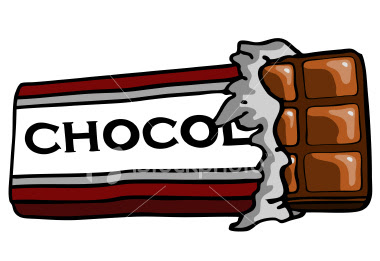

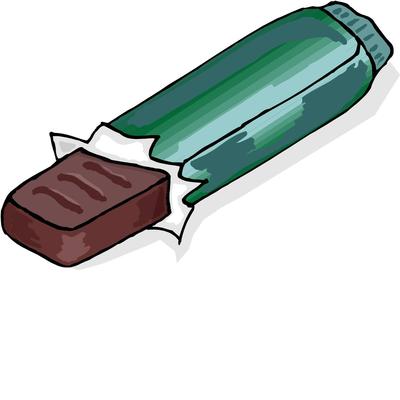

Supplement: S1 File — (DOC) [file pone.0218243.s001.doc]
