# Supplementary material for: A feasibility study with process evaluation of a teacher led resource to improve measures of child health
Source: PLoS One. 2019 Jul 2;14(7):e0218243. doi: 10.1371/journal.pone.0218243 (PMC6605653; doi:10.1371/journal.pone.0218243)
Supplement: S2 File — (DOCX) [file pone.0218243.s002.docx]

**Teacher telephone fidelity check interview guide**

Teacher phone call interviews

(Phase 1: 20 mins max)

*So, just to re-introduce myself, I am Samantha Donnelly and I am a MRes student at the university of the West of Scotland. Today, I am just phoning to see how your week has been with regards to the healthy schools resource. I understand you haven’t been familiar with the resource for too long. We’re just looking for a quick snapshot from the introductory stages of having access to the resource. Before we go any further I’d just like to let you know this call will be recorded so I make sure I don’t miss any useful information.*

*I’d like to quickly thank you for your time. Please be aware that this interview is in no way, assessing you as a teacher, or your ability to teach. We are looking to get a better understanding of the Healthy Schools resource and your honesty is something we would encourage in order for us to gauge the effectiveness of the resource accurately. This should take no longer than 20 minutes. There are no right or wrong answers and its fine if you haven’t used the resource. We are simply wanting to know whether teachers are using it or not, any issues or barriers teachers have to using it. And to find about more about your experiences of teaching health and well being topics. So that we can understand how to support teachers most effectively in the future.*

- Introduce yourself again and briefly outline the aim of the phone call/study
- **SHANARRI**

***Could you tell me if you have had the chance to cover any health and wellbeing topics this week with the children (for example SHANARRI topics of Safe, Health, Nurtured, Active etc……………….)****?*

- *What did you cover and how?*
- *What were your reasons for picking those topics?*
- *Did you use any teacher resources (for example websites, books, other teacher’s materials) which assisted you to teach these health and well-being topics?*

*If not, what were some of the key topics you were teaching this week?*

***Prompt: what were the reasons for choosing these topics?***

- *Is there anything that made it difficult for you to teach health and well-being topics this week?*
- **H.S Resource**

***Have you had the chance to use the healthy schools resource in general?***

- *What did you use it for?*
- *How did you find using it?*
- *What sections did you use and why?*
- *What sections would you say were better than others?*
- *How does using the resource compare to teaching not using it?*

*-If not, were there any barriers which restricted you from using the resource?*

Do you have another other comments about the healthy schools resource you’d like to add?

_______________________________________________

*Ok that’s great! I think I have enough information from our chat. Thanks, again for your time and don’t hesitate to contact me if you feel it would be helpful. Getting more information from you will help us to understand more about how to support teachers most effectively.*
