# Supplementary material for: A feasibility study with process evaluation of a teacher led resource to improve measures of child health
Source: PLoS One. 2019 Jul 2;14(7):e0218243. doi: 10.1371/journal.pone.0218243 (PMC6605653; doi:10.1371/journal.pone.0218243)
Supplement: S3 File — (DOCX) [file pone.0218243.s003.docx]

**Teacher post-intervention interview guide**

Face-to-face teacher interviews

To start off I’d like to let you know that this interview is being recorded to make sure I don’t miss out any useful information.

*I’d like to quickly thank you for your time. Please be aware that this interview is in no way, assessing you as a teacher, or your ability to teach. We are looking to get a better understanding of the Healthy Schools resource and your honesty is something we would encourage in order for us to gauge the effectiveness of the resource accurately. There are also a series of scenario based questions and we must stress that there are no right or wrong answers. The responses to these questions will be kept confidential within the research team and the results will be annonymised before the Healthy Schools team and the school have access to any results.*

**Opening Question**

**1. Tell me about your experiences of teaching at**

**Probe: how long have you been teaching for?**

**2. Tell me about your experiences of teaching health & well being (or the curriculum for excellence and SHANARRI) topics?**

*Probe:* How often do you teach health and wellbeing

*Probe: Do you enjoy teaching health and wellbeing? Is there anything you find challenging to teach health and wellbeing?*

***3.*** Have you used the HS Resource?

*Probe: which sections and why ?if so how often? what were the advantages/disadvantages of using HS ?*

*a) If not: what other resources if any do you use to teach health and wellbeing and why?*

4. If you could sum up the healthy schools resource in one word, what would it be?

*Probe: Why would you use this word?*

*What’s made you pick that word?*

5.How did you find using the healthy schools website page in terms of accessibility and convenience whilst teaching health and wellbeing?

6.What features of the resource, if any, were easily implemented into your teaching of health and wellbeing?

*Probe: How did you implement this into your teaching? Why was it so easy?*

*Probe: What (if any) changes you have you made to your teaching due to using the HS resource?*

6. Is the healthy schools resource something you would recommend to fellow colleagues?

1. If so, why would you recommend the resource? If not, why wouldn’t you?

*Probe:* *What could be different about the resource in order for you to want to recommend it to colleagues?*

1. What features of the resource, if any, were difficult to implement into your teaching of health and wellbeing?

*Probe: why was it difficult? What could be done to make it easier?*

1. If you could change things about the resource, if anything, what would they be?

*Probe: Why would you change this? How would you change this?*

1. Would you want to continue to use the healthy schools resource in your teaching of health and wellbeing? If so, why?
2. In relation to the HS primary topics, do you have a preference between Food and health, healthy lifestyles, PEPAS or interdisciplinary learning projects?

*Probe: What made you like this more? What could have been better about this?*

1. How would you say, if at all, the school has supported you to use the HS Resource?

*Probe: how has HS been encouraged in your school? Who does this?*

*Probe: What could be improved about the support you have been given.*

1. If not, why not and what could be changed to support your use of HS more effectively?
2. What other support, if any, do you get from the school to teach health and wellbeing in general?

*Id like to ask you a situational question in order to get an insight into day to day school environment of teaching.*

*A new teacher starts working in your school. She is recently qualified and has little experience teaching health and wellbeing. She asks you for some advice with regards to planning lessons and teaching topics. Which advice would you give her and why?*

Finally we would like to get your feedback on the research side of the study:

1. What did you think initially about participating in this study?
2. Probe: Tell me more about your experiences of being involved in this study?

*Probe: What do you think now?*

In relation to the children in your class:

1. What were your experiences of encouraging parents to consent to their child’s involvement?
2. What do you think were the children’s experiences of participating in the study
3. As a research team is there anything that would improve participation in the research study?
