# Supplementary material for: A feasibility study with process evaluation of a teacher led resource to improve measures of child health
Source: PLoS One. 2019 Jul 2;14(7):e0218243. doi: 10.1371/journal.pone.0218243 (PMC6605653; doi:10.1371/journal.pone.0218243)
Supplement: S4 File — (DOCX) [file pone.0218243.s004.docx]

| **Aim** | **Theme** | **Subtheme** | **Example quote** |
| --- | --- | --- | --- |
| **Active Ingredients of HSR use** | Design of HSR | Systematic structure of HSR | I do quite like the structure of it… I quite liked it, it is systematic… it certainly works for me, I like the suggested ideas suggested resources… I like the direction that it takes you and then I like the three areas of health and wellbeing. Teacher I |
|  |  | Curriculum relevance guidance | They have got the significant aspects of learning now across the curriculum which is really useful. Teacher J. |
|  |  | Accessible and easy to navigate | The major advantage is the layout of it and how it is organised because it makes it really easy to follow. The HSR could be used in class by displaying HSR activities and web links onto whiteboards and smart boards. Teacher J. |
|  |  |  |  |
|  |  |  |  |
|  | Facilitative school context - | Whole school approach | Well at the moment we are looking at the topics, and each one has a different focus, and that actually seems to working very well because it’s good that the whole school are doing the same things at the same time. Teacher K |
|  |  | Support from health and well-being coordinator | We’ve got a health and wellbeing co-ordinator, who has been leading the drive on using the Healthy Schools resource. She’s been great in just keeping everybody on, this is our focus this month and different support and she’s really available if you have any questions or anything you’re unsure of. Teacher I |
|  |  | Evening development sessions | Well at the CAT night, we all sat down, the health and wellbeing coordinator showed us it (the Healthy Schools Resource) and then we spoke about the resource and the planners that we use, and we spoke about resources in the school that we can use. We worked in groups and looked through the levels and then progression in school and what they get taught at each stage, that kind of thing, which was really useful to me having no experience. Teacher K |

**Qualitative data**

**Table A. Teacher interview data: The active ingredients of teacher’s use of the HSR**

**Table B. Teacher interview data: The perceived outcomes of using HSR and research participation for teachers and pupils**

| **Aim** | **Theme** | **Subtheme** | **Example quote** |
| --- | --- | --- | --- |
| **Teacher outcomes** | Outcomes of using HSR | Saved planning time | It has been really good because it like lays out exactly kind of the best thing is when you go on you are starting to plan… and that just can save so much time when it comes to planning so.. Teacher B |
|  |  | More varied health and well being classes | There is much more variety to the lessons that there has been before. Teacher J |
|  |  | Teaching health topics more | I would say I definitely teach health and wellbeing more often than I did before. Teacher J |
|  |  | Less likely to skip teaching health topics | In the past I have found it boring to plan for, boring to teach and you would sort of skip past it, that is what ends up happening because you are always tight for time… So the fact that it changes every month keeps it fresh. Teacher J |
|  | Outcomes of research participation | Self-evaluation and reflection regarding teaching practice | We’re always talking about self-evaluation and whatever but we don’t necessarily, well I've not really necessarily thought about it in this area… I think it's given us a chance to think about how we do teach and what we use to teach it. So it's like self-evaluation for us, which I think is quite good. Teacher K |
|  |  | Children discussing HWB more | So it got them actually talking about it more than probably than they might have done before, though previously it was more discrete topic area covered in a set month or a set term. Teacher F |
| **Pupil outcomes** | Outcomes of using HSR | More comprehensive child support | I think that’s so important with health and wellbeing and to ensure that the children are gaining a wealth of knowledge throughout the wellbeing indicators, just to ensure that they have – they’re informed that they have to deal with – getting it right for every child, to ensure that the all the whole child’s needs are met. And I think through the wellbeing indicators that’s achievable. Teacher H |
|  |  | Monthly focus Embeds knowledge | Because you say, remember, this month is respecting, it about respecting other people, and you’re talking about that constantly, so it’s really embedded with the children. Teacher F |
| **School Outcomes** | Outcomes of using HSR | Creates a sense of community | That creates a nice sense of community, if you like, and I think that is really important with health and wellbeing too, it should not just be that is a lesson and that is it. It should be something that we are all aware, that we are all trying to do together, like part of your life basically. Teacher J |

**Table C. Teacher interview data: recommendations to improve the HSR**

| **Aim** | **Theme** | **Subtheme** | **Example quote** | |
| --- | --- | --- | --- | --- |
| **HSR changes** | Functionality | Copy & Paste | I know there’s no copy and paste function and that is a bit frustrating because it is just the way the world is today that people copy and paste from everywhere. Teacher J |  |
|  |  | Editing content | Even if they were online and editable, that could take the frame and tailor it to what you’re wanting. Teacher E |  |
|  |  | Access to HSR website via search engines | The only thing is you couldn’t Google it. It brought up too many when you put in healthy. Teacher C |  |
|  |  | Website navigation using a search bar | I mean I think it took me a wee while to go find it and then find my way around. Teacher I  Maybe if there was some sort of search on it where you could just put up your term in and it could maybe flag up the bit. Teacher A |  |
|  | Content | Standardised templates | Maybe another thing that would be beneficial is to upload more templates for things. Again, every teacher across South and North Lanarkshire will be creating a template. So why have hundreds of them when there could be one good one out there? Teacher J |  |
|  |  | Additional health topics | As a teacher, you could say, let’s talk about our bodies during respected and then link it to what’s good and bad… but I don’t think that’s set out. Teacher I |  |
|  |  | More assessment tools | Some sort of assessment tool would be good to have because that is always a big question from management is how are we assessing the children and how are we able to see their progress? I know there is tracking grids but certain people may argue that okay that tracks it but have you really got an in-depth assessment. Teacher J |  |
|  | HSR Training | More introductory training | I would've needed time with the resource actually using it to learn how to use it... even if it's a couple of hours actually at a computer with someone... I learn better that way... It was a very quick demonstration of how to use it. Teacher D |  |

**Table D. Teacher interview data: teacher perceptions of research feasibility**

| **Aim** | **Theme** | **Subtheme** | **Example quote** |
| --- | --- | --- | --- |
| **Teacher experiences of participation** | Commitment | Manageable workload | Yeah, I don't think that anything's been like added to my workload really. Teacher A |
|  | Recruitment | Start at the beginning of the academic year | If it was given to me at the beginning of the year, when I came into a school, they said, right, here's a resource that you can use, I would probably look at it and that's when I would look at what resources I'm going to use. Teacher D |
|  |  | Use a teacher advocate | With teachers, it just takes one person to volunteer and then everyone will volunteer. Teacher D |
| **Increasing pupil recruitment** | Parent information | Parents evenings | Whether or not you did the parent night thing they would still be able to understand what it is and they could still make that a kind of more informed decision whereas I think possibly a lot of people might have said no because they didn’t know what the blood was or weren’t sure about the relevance of that or whatever. Teacher E |
|  |  | Demonstrations  Teacher-led talks | Maybe a demonstration for the parents of what’s going to happen to the children. Teacher E  And I don't know if we could maybe have if we’d had the chance to maybe talk to them about it? To see what it was that maybe you’d have got more responses. Teacher K |
|  | Pupil encouragement | Posters | That somehow bright, colourful, that maybe got the message going across is like being, just trying to think not a scientist, but something like that…Teacher F |
|  |  | Rewards | Did they get a sticker or anything? . . . I would be thinking of a wee something they can go away with, even a certificate to say I did this… Where it could be said at the beginning to motivate the children… But if you are always dealing with children a big draw for anything is will I get anything at the end to say I’ve done this that I can put on my wall. Teacher F |
